# The potential causal effect of the pre-pregnancy dietary phytochemical index on gestational diabetes mellitus: a prospective cohort study

**DOI:** 10.1186/s12884-024-06643-4

**Published:** 2024-06-28

**Authors:** Neda Heidarzadeh-Esfahani, Javad Heshmati, Reihaneh Pirjani, Ashraf Moini, Mehrnoosh shafaatdoost, Mahnaz Esmaeili, Azar Mardi-Mamaghani, Seyyed Mostafa Nachvak, Mahdi Sepidarkish

**Affiliations:** 1 Nutritional Sciences Department, School of Nutrition Sciences and Food Technology, Kermanshah University of Medical Sciences, Kermanshah, Iran; 2 University of Ottawa Heart Institute, University of Ottawa, Ottawa, Canada; 3 Department of Obstetrics and Gynecology, Arash Women’s Hospital, Tehran University of Medical Sciences, Tehran, Iran; 4 Department of Endocrinology and Female Infertility, Reproductive Biomedicine Research Center, Royan Institute for Reproductive Biomedicine, ACECR, Tehran, Iran; 5 Department of Clinical Nutrition, School of Nutritional Sciences and Dietetic, Tehran University of Medical Sciences, Tehran, Iran; 6 Department of Genetics, Reproductive Biomedicine Research Center, Royan Institute for Reproductive Biomedicine, ACECR, Tehran, Iran; 7 Department of Andrology, Reproductive Biomedicine Research Center, Royan Institute for Reproductive Biomedicine, ACECR, Tehran, Iran; 8 Population, Family and Spiritual Health Research Center, Health Research Institute, Babol University of Medical Sciences, Babol, Iran

**Keywords:** Dietary phytochemical index, Gestational diabetes mellitus, DPI, GDM

## Abstract

**Background:**

Phytochemicals are non-nutritive bioactive compounds with beneficial effects on the metabolism of glucose. This study aimed to clarify the possible causal effect of the pre-pregnancy dietary phytochemical index (DPI) on gestational diabetes mellitus (GDM).

**Methods:**

In this prospective cohort study 1,856 pregnant women aged 18–45 years who were in their first trimester, were recruited and followed up until delivery. The dietary intakes of participants were examined using an interviewer-administered validated 168-item semi-quantitative food frequency questionnaire (FFQ). Inverse probability weighting (IPW) of propensity scores (PS), estimated from the generalized boosted model (GBM) were used to obtain a adjusted risk ratio (aRR) for potential confounders.

**Results:**

During the follow-up period, 369 (19.88%) women were diagnosed with GDM. DPI scores ranged from 6.09 to 89.45. There was no association between DPI scores and GDM (aRR: 1.01, 95% confidence interval [CI]: 0.92, 1.08; p trend = 0.922). When comparing DPI quartile 4 (most pro-phytochemical content) to quartile 1 (few phytochemical contents), there was no significant difference between them (aRR: 0.97; 95% CI: 0.75, 1.25; *p* = 0.852). Also, there was no significant difference between DPI quartile 3 and quartile 1 (aRR: 1.04; 95% CI: 0.81, 1.34; *p* = 0.741) as well as DPI quartile 2 and quartile 1 (aRR: 0.92; 95% CI: 0.71, 1.21; *p* = 0.593).

**Conclusions:**

Although this data did not support the association between pre-pregnancy DPI scores and GDM, further cohort studies to ascertain the causal association between them are warranted.

## Background

Women with GDM experience glucose intolerance in the second or third trimester of pregnancy without any clear manifest diabetes before pregnancy [1]. GDM prevalence was reported to range from 9.3 to 25.5% (average 17.8%) and the prevalence has been increasing worldwide [2, 3]. GDM is associated with an increased risk for many short-term and long-term consequences for both mother and offspring, including obesity, impaired glucose metabolism, and cardiovascular disease [4–8]. Thus, it is important to come up invaluable approach for GDM prevention and management.

There is now substantial evidence that maternal dietary patterns before and during pregnancy prevent or delay the development of GDM [9–12] however, the focus has been on identifying crucial risk factors during pregnancy. Recently, an increasing interest has emerged in the worthwhile effects of plant-based dietary patterns and phytochemical plant-derive bioactive compounds for the management of GDM [13, 14].

Phytochemicals are biologically non-nutritive bioactive compounds divided into several classes, including: Alkaloids, Glycosides, organosulfur compounds (thiosulfinate and isothiocyanates) phenolic compounds (flavonoids, phenolic acids, hydroxycinnamic acids, lignans, polyphenols, and stilbenoids), tannins, Terpenes, saponins, Anthraquinones, essential oils, and steroids [15, 16].

The DPI, which was proposed and developed for the first time by McCarty, is determined according to the percent of daily energy intake derived from phytochemical-rich foods such as fruits, vegetables, legumes, whole grains, nuts, seeds, soy products, juices (fruit and vegetable), and other plant foods [17].

DPI have been inversely associated with risk of cardiovascular disease [18, 19], insulin resistance [20], metabolic syndrome [21], and cancer [21]. Plausible mechanisms underlying causes of beneficial traits of phytochemicals on non-communicable diseases are antioxidant and anti-inflammatory effects, enhanced glycemic control, regulated body weight, improved insulin sensitivity, and gut microbiota [22–24].

Limited observational studies have investigated associations between DPI and the improvement of glucose tolerance and insulin sensitivity [20, 25]. According to our review, there is no study presenting the association between DPI and GDM. Denoting the association between DPI and glycemic indices among affected women may provide the obvious starting point for GDM prevention and treatment. Hence, in the present study, we aimed to determine the possible causal effect of the pre-pregnancy DPI on GDM.

## Methods

### Study design and participants

We conducted a prospective cohort study - Mothers and their children’s health (MATCH) study at the Arash Women’s Hospital in Tehran, Iran between February 2020 and January 2023. The details of this study and further information on methods have been described previously [26]. The MATCH protocol was approved by the institutional review boards of the Tehran University of Medical Sciences (Protocol number: IR.TUMS.MEDICINE.REC.1398.576).

Briefly, the pregnant women aged from 18 to 45 years who were at less than 12 weeks of gestation, and attending antenatal care in Arash Women’s Hospital in Tehran were included between February 2020 and August 2021. Furthermore, women who reported a previous diagnosis of metabolic or chronic diseases, following a special diet, using certain food supplements (except for pregnancy supplements such as iron or folate), suffering from physical, mental, cognitive disability, and having an unusual total energy intake (< 800 or > 4200 kcal/day), were excluded from the current study. Total daily energy intake by summing up the calories from all food items were reported in 168-item semi-quantitative food frequency questionnaire (FFQ).

### Data collection

Ten trained observers completed a structured questionnaire through face-to-face interviews to obtain sociodemographic, history of underlying disease, and lifestyle variables, including smoking, alcohol, dietary pattern, physical activity, and sleep quality pre-pregnancy and early pregnancy. The quantity and quality of physical activity was assessed by the International Physical Activity Questionnaire (IPAQ) using Metabolic Equivalent of Tasks (METs) [26]. Also, the anthropometric indices, including weight, height, waist, and hip circumferences were measured by our trained staff accurately.

### Dietary intakes assessment

In the first visit, dietary intake was evaluated by using an interviewer-administrated 168-item FFQ that contains questions about the type/brand, cooking methods, frequency, and the amount of all foods and drinks they consumed during the one-year leading up to the pregnancy. The validity and reliability of FFQ were confirmed in the Tehran Lipid and Glucose Study (TLGS) in Iran [27]. For the FFQ data, portion sizes will be converted to grams per week per food item by two experienced nutritionists.

### Exposure assessment

The DPI was determined based on the method developed by McCarty; [PI = (daily energy derived from phytochemical-rich foods (kcal)/total daily energy intake (kcal)) × 100] [17]. Fruits and vegetables (except potatoes), legumes, whole grains, nuts, soy products, olives, and olive oil were categorized into phytochemical-rich foods. Natural fruit and vegetable juices such as tomato sauces were included in the fruit and vegetable groups due to their high phytochemical content.

### Outcome assessment

Screening and diagnosis of GDM were carried out according to the results of the one-step method which includes a fasting glucose test followed by a 75-gram, 2-hour diagnostic oral glucose tolerance test (OGTT) between 24 and 28 weeks of gestation. Using the one-step method, women were considered to have screened positive for GDM if they had a serum glucose value fasting ≥ 92 mg/dl, 1-hour ≥ 180 mg/dl, and 2-hour ≥ 153 mg/dl [28].

### Statistical analysis

**Fig. 1:**
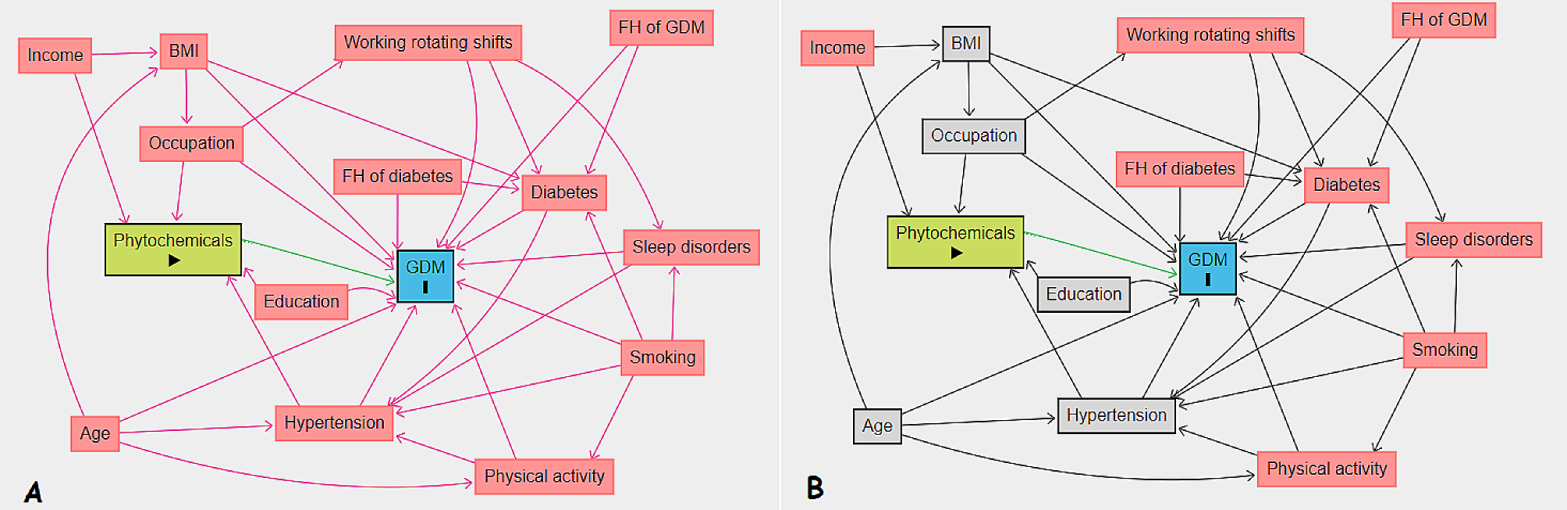
Directed acyclic graphs, A: Unadjusted, B: Adjusted. BMI, Body Mass Index; GDM, Gestational Diabetes Mellitus; FH, Family History

We presented continuous baseline characteristics as mean (± SD) or median (interquartile range, IQR) and compared using one-way analysis of variance and independent t-test. Also, we expressed categorical variables as numbers (percentages) and compared using Chi-square test. We used multiple imputations based on chained equations, which fill in missing values in multiple variables iteratively using a sequence of univariate imputation models with a fully conditional specification of prediction equations. We used the generalized boosted model (GBM) for the estimation of participants’ propensity scores for DPI, so that covariate imbalance between the exposed (quartiles 1–3 (Q1–Q3) for DPI) and non-exposed groups was minimized. We employed ‘TWANG’ package to estimate propensity scores using an automated, nonparametric machine learning method, and generalized boosted models based on 10,000 regression trees. We selected the minimal sufficient variables using directed acyclic graphs (DAGs), based on the web tool dagitty.net (Fig. 1) [29]. We evaluated the association between DPI and the incidence of GDM by calculating adjusted risk ratios (aRRs) and corresponding 95% confidence intervals based on the weighted modified Poisson regression with the inverse probability weight (IPTW). In addition, we stratified the analysis based on age to determine whether the risk of GDM affected by it. The data processing and statistical analysis were performed using the Stata statistical package version 17 (Stata Corp LP, College Station, TX, USA) and R statistical software (Version 4.2.1; The R Foundation for Statistical Computing, Vienna, Austria).

## Results

**Fig. 2:**
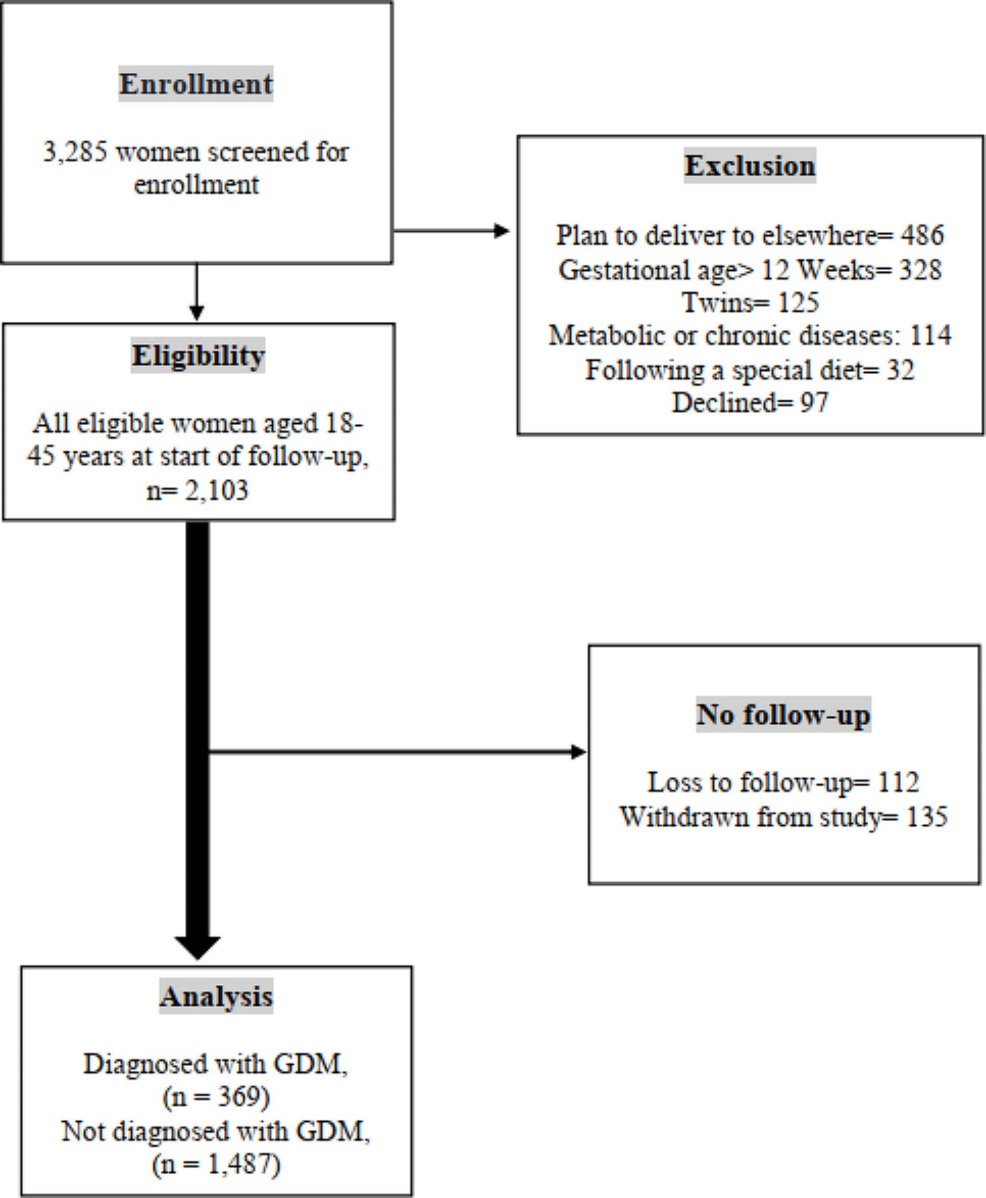
Flow Diagram of Study Participants. † Gestational Diabetes Mellitus; flow diagram showing participant recruitment from enrolment to corresponding numbers of women who were and were not diagnosed with gestational diabetes. Reasons and number of exclusions are stated accordingly

The flow diagram in Fig. 2 depicts the number of pregnant women examined at each time point, as well as those lost to follow-up and the reasons for dropout. A total of 3,285 women were enrolled from 1 February 2020 and January 2023. Based on initial screening, 2,103 women were eligible for inclusion in the study, of whom 1,856 had complete and 247 (11.74%) had incomplete follow-up data. We excluded 1,182 participants for the following reasons: (І) plan to deliver elsewhere (*n* = 486, 41.11%); (Π) gestational age > 12 Weeks (*n* = 328, 27.74%); (III) multiple pregnancies (*n* = 125, 10.57%); (IV) metabolic or chronic diseases (*n* = 114; 9.64%); (V) following a special diet (*n* = 32; 2.70%); and (VI) declined (*n* = 97, 8.20%) (Fig. 2).

The demographic and clinical characteristics of women with and without GDM as well as across quartiles of DPI are presented in Tables 1 and 2, respectively. At the study baseline, the mean age and BMI of the included women were 32.9 ± 6.1 years and 25.9 ± 8.3 kg/m2, respectively. 7.5% (*n* = 149) of study participants were employed and 45.5% (*n* = 845) had an academic education. During the follow-up period, 369 (19.88%) women were diagnosed with GDM. Women with GDM were older (34.8 ± 5.7 vs. 32.4 ± 5.9; *p* < 0.001), heavier (67.9 ± 13.1 vs. 65.2 ± 12.4; *p* < 0.001), more likely to be current smokers (90/369, 24.4% vs. 278/1,487, 18.7%; *p* = 0.140), pre-existing diabetes (83/369, 22.5% vs. 106/1,487, 7.1%; *p* < 0.001), and had a higher frequency of family history of diabetes than controls (174/369, 47.1% vs. 572/1,487, 38.5%; *p* = 0.002).

**Table 1:** Baseline characteristics of non-pregnant women according to prospective development of GDM (*n* = 1,856)

	GDM incidence (*n* = 369)	No GDM (*n* = 1,487)		*p*-value
Age (year)^a^	34.83 ± 5.74	32.46 ± 5.96		< 0.001
Employment ^b^				
Housewife	330 (89.43)	1,385 (93.27)		0.012
Employed	39 (10.57)	100 (6.73)		
Education status ^b^				
Junior high school or lower	103 (27.91)	349 (23.50)		0.176
Senior high school	156 (42.28)	689 (46.40)		
Undergraduate college or higher	110 (29.81)	447 (30.10)		
Pre-pregnancy BMI (kg/m^2^)^a^	26.84 ± 8.56	25.75 ± 8.31		0.024
Pre-pregnancy weight ^a^	67.93 ± 13.08	65.27 ± 12.42		< 0.001
Gravidity ^a^	1.25 ± 1.07	1.10 ± 1.08		0.021
Parity ^a^	0.92 ± 0.83	0.80 ± 0.81		0.013
Live birth ^a^	0.77 ± 0.78	0.87 ± 0.80		0.033
Miscarriage ^a^	0.03 ± 0.18	0.03 ± 0.17		0.629
Smoking ^b^				
Current smoker	90 (24.39)	278 (18.72)		0.083
Before smoker	14 (3.79)	77 (5.19)		
Passive smoker	19 (5.15)	79 (5.32)		
Non-smoker	246 (66.67)	1,051 (70.77)		
Waist circumference (cm) ^a^	98.87 ± 12.97	96.57 ± 12.74		0.002
Hip circumference (cm) ^a^	107.68 ± 9.85	106.31 ± 10.10		0.019
Waist/ Hip ratio ^a^	0.91 ± 0.08	0.90 ± 0.09		0.066
Pre-existing hypertension ^b^	15 (4.07)	48 (3.23)		0.429
Pre-existing diabetes ^b^	83 (22.49)	106 (7.14)		< 0.001
Pre-existing GDM ^b^	40 (10.84)	81 (5.45)		0.570
Family history of DM ^b^	174 (47.15)	572 (38.52)		0.002
Dietary caloric intake (kcal/day) ^b^				
≤ 2300	161 (43.87)	628 (42.35)		0.597
> 2300	206 (56.13)	855 (57.65)		
Physical activity (MET-h/week) ^c^	2692 (1260, 5295)	2919 (1458, 4942)		0.779

BMI: body mass index, DM: diabetes mellitus, GDM: gestational diabetes mellitus. ^a^ Values given as mean ± SD (standard deviation) and analyzed by independent t-test. ^b^ Values given as numbers (percentage) and analyzed by Chi-squared test or Fisher’s exact test. ^C^ Values are given as median (IQR) and analyzed by independent t-test

Missing data: age, *n* = 10 (0.5%); occupation, *n* = 1 (0.1%); education, *n* = 7 (0.4%); smoking, *n* = 11 (0.6%); history of GDM, *n* = 1 (0.1%); family history of diabetes, *n* = 3 (0.2%); parity, *n* = 56 (3%); BMI, *n* = 28 (1.5%); dietary caloric intake, *n* = 4 (0.2%); physical activity, *n* = 83 (4.5%)

**Table 2:** Characteristics of participants by categories of the dietary phytochemical index (*n* = 1,856)

Pre-pregnancy characteristic	Quartile 1	Quartile 2	Quartile 3	Quartile 4	*p*
Age (year)^a^	31.94 ± 6.16	32.58 ± 5.76	33.17 ± 5.79	34.04 ± 6.06	< 0.001
Employment ^b^					
Housewife	429 (92.46)	430 (92.87)	423 (91.36)	432 (93.30)	0.707
Employed	35 (7.54)	33 (7.13)	40 (8.64)	31 (6.70)	
Education status ^b^					
Junior high school or lower	114 (24.57)	116 (25.05)	111 (23.97)	111 (23.97)	0.364
Senior high school	228 (49.14)	213 (46.01)	201 (43.41)	202 (43.63)	
Undergraduate college or higher	122 (26.29)	134 (29.94)	151 (32.62)	150 (32.40)	
Pre-pregnancy BMI (kg/m^2^)^a^	26.32 ± 11.68	25.88 ± 8.63	25.44 ± 4.97	26.23 ± 6.69	0.363
Pre-pregnancy weight ^a^	64.51 ± 12.45	65.57 ± 12.06	66.02 ± 13.09	67.11 ± 12.69	0.017
Gravidity ^a^	1.04 ± 0.95	1.10 ± 1.01	1.12 ± 1.11	1.27 ± 1.21	0.010
Parity ^a^	0.82 ± 0.78	0.76 ± 0.71	0.79 ± 0.80	0.93 ± 0.94	0.007
Live birth ^a^	0.77 ± 0.76	0.74 ± 0.71	0.77 ± 0.77	0.88 ± 0.87	0.040
Miscarriage ^a^	0.02 ± 0.14	0.04 ± 0.21	0.03 ± 0.17	0.03 ± 0.18	0.728
Smoking ^b^					
Current smoker	110 (23.71)	95 (20.52)	91 (19.65)	71 (15.33)	0.271
Before smoker	21 (4.53)	24 (5.18)	22 (4.75)	24 (5.18)	
Passive smoker	24 (5.17)	25 (5.40)	26 (5.62)	23 (4.97)	
Non-smoker	309 (66.59)	319 (68.90)	324 (69.98)	345 (74.51)	
Waist circumference (cm) ^a^	96.88 ± 12.18	96.79 ± 12.31	97.10 ± 13.52	97.33 ± 13.27	0.917
Hip circumference (cm) ^a^	105.79 ± 9.94	106.80 ± 10.17	106.21 ± 10.07	107.53 ± 10.01	0.048
Waist/ Hip ratio ^a^	0.91 ± 0.08	0.90 ± 0.09	0.91 ± 0.10	0.90 ± 0.08	0.146
Pre-existing hypertension ^b^	12 (2.59)	11 (2.38)	21 (4.54)	19 (4.10)	0.177
Pre-existing diabetes ^b^	36 (7.76)	36 (7.78)	50 (10.80)	67 (14.47)	0.002
Pre-existing GDM ^b^	22 (4.74)	24 (5.18)	33 (7.13)	42 (9.07)	0.030
Family history of DM ^b^	168 (36.21)	188 (40.60)	184 (39.74)	206 (44.49)	0.002
Dietary caloric intake (kcal/day) ^b^					
≤ 2300	272 (58.75)	202 (43.72)	163 (35.21)	152 (32.97)	< 0.001
> 2300	191 (41.25)	260 (58.28)	300 (64.79)	309 (67.03)	
Physical activity (MET-h/week) ^c^	2904 (1367, 5448)	2793 (1408, 4917)	2692 (1326, 5097)	2994 (1611, 4818)	0.796

BMI: body mass index, DM: diabetes mellitus, GDM: gestational diabetes mellitus. ^a^ Values given as mean ± SD (standard deviation) and analyzed by independent t-test. ^b^ Values given as a number (percentage) and analyzed by Chi-squared test or Fisher’s exact test. ^C^ Values are given as median (IQR) and analyzed by independent t-test. Missing data: age, *n* = 10 (0.5%); occupation, *n* = 1 (0.1%); education, *n* = 7 (0.4%); smoking, *n* = 11 (0.6%); history of GDM, *n* = 1 (0.1%); family history of diabetes, *n* = 3 (0.2%); parity, *n* = 56 (3%); BMI, *n* = 28 (1.5%); dietary caloric intake, *n* = 4 (0.2%); physical activity, *n* = 83 (4.5%);

The DPI score of the women’s diet ranged from 6.1 to 89.4 with a median (IQR) of 40.3 (19.8). Also, the DPI score of women across quartile categories in the first, second and third quartiles was 30.9, 40.3, and 50.8, respectively. Pregnant women in the highest quartile had a higher frequency of pre-existing diabetes, GDM, and a family history of diabetes. Also, they had a higher pre-existing BMI and dietary caloric intake (kcal/day). The overall mean DIP in the women with and without GDM were 41.5 ± 13.6 and 41.1 ± 13.8, respectively (*p* = 0.614).

We outlined the crude and multivariate-adjusted risk ratios (aRRs) for the association between DPI and GDM in Table 3. We found no significant association between DPI and GDM in the crude model (crude RR: 1.03, 95% CI: 0.95, 1.12, *p* = 0.413). This association remained non-significant after adjustment for potential confounders, including body mass index (kg/m2), occupation, age, hypertension, education, and gastrointestinal diseases (aRR: 1.01, 95% CI: 0.92, 1.08, *p* = 0.922). The crude RR of GDM in a quartile with the highest DPI scores (Q4), compared to that with the lowest scores (Q1), was 1.06 (95% CI: 0.82, 1.38, *p* = 0.612). After additional adjustment for potential confounders, including body mass index (kg/m2), occupation, age, hypertension, education, and gastrointestinal diseases, associations were attenuated but remained non-significant (aRR: 0.97, 95% CI: 0.75, 1.25, *p* = 0.852).

**Table 3:** Relative risks (95% CIs) for associations between dietary phytochemical index at baseline and incidence of GDM (*n* = 1,856)

Quantile of dietary pattern scores	GDM cases *n* (% pregnancies)	Model 1		Model 2	
		cRR (95% CI)	**p**	aRR (95% CI)	**p**
Quartile 1	90 (19.40)	1 (reference)		1 (reference)	
Quartile 2	85 (18.36)	0.94 (0.72, 1.23)	0.687	0.92 (0.71, 1.21)	0.593
Quartile 3	98 (21.17)	1.09 (0.84, 1.40)	0.504	1.04 (0.81, 1.34)	0.741
Quartile 4	96 (20.73)	1.06 (0.82, 1.38)	0.612	0.97 (0.75, 1.25)	0.852
Continuous ^a^	369 (100)	1.03 (0.95, 1.12)	0.413	1.01 (0.92, 1.08)	0.922

Model 1 was a univariate model; model 2 was a multivariate model to account for potential confounding, the impact of the dietary phytochemical index on gestational diabetes mellitus was estimated using a propensity score approach. We used an inverse probability weighting estimator to estimate the average treatment effect. The propensity score was estimated using a gradient-boosting algorithm. The following variables were included in the propensity score model: body mass index (kg/m2), occupation, age, hypertension, education, and gastrointestinal diseases. Minimal sufficient adjustment sets were selected based on the directed acyclic graph (presented in Fig. 1)

^each^ 1 SD increase in score

cRR, crude risk ratio; aRR, adjusted risk ratio

In stratified analyses, we studied whether the effects of DFI on GDM could be modified by age. In these analyses, the main analyses showed similar results by age categories. We found no significant association between DPI and GDM in all age categories (Tables 4, 5 and 6).

**Table 4:** Risk ratios (95% CIs) for associations between dietary phytochemical index at baseline and incidence of GDM in women under 25 years old (*n* = 224)

Quantile of dietary pattern scores	GDM cases *n* (% pregnancies)	Model 1		Model 2	
		cRR (95% CI)	**p**	aRR (95% CI)	**p**
Quartile 1	11 (13.41)	1 (reference)		1 (reference)	
Quartile 2	5 (8.62)	0.64 (0.23, 1.76)	0.391	0.66 (0.23, 1.85)	0.435
Quartile 3	5 (11.36)	0.84 (0.31, 2.30)	0.745	0.86 (0.31, 2.35)	0.774
Quartile 4	2 (5.01)	0.37 (0.08, 1.63)	0.190	0.37 (0.08, 1.68)	0.200
Continuous ^a^	23 (100)	0.78 (0.54, 1.14)	0.214	0.79 (0.54, 1.15)	0.223

Model 1 was a univariate model; model 2 was a multivariate model to account for potential confounding, the impact of the dietary phytochemical index on gestational diabetes mellitus was estimated using a propensity score approach. We used an inverse probability weighting estimator to estimate the average treatment effect. The propensity score was estimated using a gradient-boosting algorithm. The following variables were included in the propensity score model: body mass index (kg/m2), occupation, age, hypertension, education, and gastrointestinal diseases. Minimal sufficient adjustment sets were selected based on the directed acyclic graph (presented in Fig. 1)

^each^ 1 SD increase in score

cRR, crude risk ratio; aRR, adjusted risk ratio

**Table 5:** Risk ratios (95% CIs) for associations between dietary phytochemical index at baseline and incidence of GDM in women between 25–35 years old (*n* = 996)

Quantile of dietary pattern scores	GDM cases *n* (% pregnancies)	Model 1		Model 2	
		cRR (95% CI)	**p**	aRR (95% CI)	**p**
Quartile 1	46 (18.32)	1 (reference)		1 (reference)	
Quartile 2	47 (18.35)	1.01 (0.69, 1.44)	0.992	1.01 (0.69, 1.45)	0.977
Quartile 3	44 (16.85)	0.91 (0.63, 1.33)	0.663	0.91 (0.62, 1.33)	0.635
Quartile 4	37 (16.37)	0.89 (0.60, 1.32)	0.575	0.89 (0.60, 1.33)	0.601
Continuous ^a^	174 (100)	0.95 (0.84, 1.08)	0.500	0.95 (0.84, 1.08)	0.510

Model 1 was a univariate model; model 2 was a multivariate model to account for potential confounding, the impact of the dietary phytochemical index on gestational diabetes mellitus was estimated using a propensity score approach. We used an inverse probability weighting estimator to estimate the average treatment effect. The propensity score was estimated using a gradient-boosting algorithm. The following variables were included in the propensity score model: body mass index (kg/m2), occupation, age, hypertension, education, and gastrointestinal diseases. Minimal sufficient adjustment sets were selected based on the directed acyclic graph (presented in Fig. 1)

^each^ 1 SD increase in score

cRR, crude risk ratio; aRR, adjusted risk ratio

**Table 6:** Risk ratios (95% CIs) for associations between dietary phytochemical index at baseline and incidence of GDM in women higher 35 years old (*n* = 636)

Quantile of dietary pattern scores	GDM cases *n* (% pregnancies)	Model 1		Model 2	
		cRR (95% CI)	**p**	aRR (95% CI)	**p**
Quartile 1	32 (24.80)	1 (reference)		1 (reference)	
Quartile 2	33 (22.14)	0.89 (0.58, 1.36)	0.603	0.90 (0.58, 1.38)	0.638
Quartile 3	49 (30.81)	1.24 (0.84, 1.81)	0.265	1.29 (0.88, 1.89)	0.189
Quartile 4	57 (28.93)	1.16 (0.80, 1.69)	0.419	1.19 (0.82, 1.73)	0.341
Continuous ^a^	171 (100)	1.08 (0.96, 1.21)	0.186	1.09 (0.97, 1.22)	0.138

Model 1 was a univariate model; model 2 was a multivariate model to account for potential confounding, the impact of the dietary phytochemical index on gestational diabetes mellitus was estimated using a propensity score approach. We used an inverse probability weighting estimator to estimate the average treatment effect. The propensity score was estimated using a gradient-boosting algorithm. The following variables were included in the propensity score model: body mass index (kg/m2), occupation, age, hypertension, education, and gastrointestinal diseases. Minimal sufficient adjustment sets were selected based on the directed acyclic graph (presented in Fig. 1)

^each^ 1 SD increase in score

cRR, crude risk ratio; aRR, adjusted risk ratio

## Discussion

The purpose of the current study was to evaluate the causal effect of calorie intake from phytochemical-rich foods on GDM, using propensity score to minimize potential confounding factors. In this prospective study, after accounting for non-dietary covariates such as body mass index (kg/m2), occupation, age, hypertension, education, and gastrointestinal diseases, the association between the higher load of calorie intake from phytochemical-rich foods and occurrence of GDM was found to be nonsignificant after a 9-months follow-up in pregnant women.

To our knowledge, this work was the first study observed the association between DPI and GDM. However, some studies have examined the association between DPI and glucose homeostasis disruption which showed controversial results [20, 25, 30–33]. For instance, the finding of mentioned prospective study are aligned with some observational studies on DPI and hyperglycemia. In a cross-sectional investigation on 2,326 adults aged between 20 and 70 years which aimed to investigate the association between DPI and metabolic syndrome, no significant association was yielded between DPI and the prevalence of high serum FBS in crud and full adjustments model [33]. Firouzabadi et al. conducted a cross-sectional study reported no significant association between odds of hyperglycemia in men and women across quartiles of DPI in both crude model and after adjusting for age, energy intake, marital status, educational status, occupation physical activity, and smoking status [31].

In stark contrast, however, in the Tehran Lipid and Glucose cohort study across 1,141 participants with an average of three years of follow-up, showed considerably a reduction risk of insulin insensitivity (OR = 0.11, 95% CI: 0.05, 0.24), insulin resistance (OR = 0.48, 95% CI: 0.25, 0.93) and hyperinsulinmia (OR = 0.14, 95% CI: 0.07, 0.25) in higher quartiles of DPI after adjustment for non-dietary factors [20]. The potential protective impact of phytochemicals is attributed to their antioxidant properties, enhancement of beta cell function, promotion of insulin response, and reduction of glucose-dependent insulinotropic polypeptide (GIP) and glucagon-like polypeptide-1 (GLP-1) levels. These mechanisms are considered key in the pathophysiological effects of phytochemicals [20]. In agreement with this finding, Delshad Aghdam et al. found that the risk of hyperglycemia significantly decreased by 88% (OR = 0.12, 95% CI: 0.02, 0.82) after adjusted for age, sex, total energy intake (kcal/day), physical activity (MET/min/week), BMI (kg/m2), diabetes duration (year), total insulin dose (unit/day), education and dietary supplement intake in participants with T1DM in the highest tertile of DPI [30]. Moreover, the case-control study which denoted a high level of DPI score is related to a lower risk of prediabetes (OR = 0.09, 95% CI: 0.03, 0.25). Also this study showed individuals in the higher quartiles of DPI had significantly lower FBG and OGTT (p-trend < 0.001) [25]. In contrast, we did not observe any statistically significant association between DPI and OGTT in women with GDM.

In addition, in a case-control study with 210 diabetic women, a significant negative association of DPI with FBS (*p* = 0.04) was observed in the case group with diabetic nephropathy [32].

The fact that studies are inconsistent might be due to differences in sample size, methodology, different dietary intake assessment, and eligibility criteria (most of them excluded pregnant women).

It is worth noting that in this study we used DPI which is practically useful to induce synergetic clinical functions of phytochemicals isolated from various types of foods and it can bring in its wake modulating physiologically [17]. By contrast, the majority of findings from prior studies can be drawn from certain phytochemicals and their effects on GDM.

The results of the present study are in line with the findings of a longitudinal cohort study conducted on pregnant with twins in China which indicated that no significant association was shown between the risk of GDM and vegetables and fruit-based pattern [34].

Our findings is in accordance with a recent meta-analysis of 12 epidemiological studies revealed that there was not any significant interplay between consuming polyphenol-rich fruits, seeds, and whole grains with GDM, nonetheless, the highest adherence to the Mediterranean diet (MedDiet) associated with lower risk of GDM [14]. Meanwhile, the inverse association of MedDiet with GDM has been appraised in another systematic review and meta-analysis of observational studies [35]. MedDiet is associated with better control of lipid and glycemic profiles [36–38], and eventually lower incident risk in type 2 diabetes [39]. As a matter of fact, the protection effect of MedDiet was mainly manifested via its content of poly and mono-unsaturated fatty acids by modulating inflammatory processes [40]. Moreover, the above-mentioned studies were mainly conducted in Western and American countries.

On the other hand, two prospective studies have presented the association between whole grain and the risk of GDM [41, 42] but with inconsistent findings. In a prospective cohort study in China, a whole grain-sea food pattern was associated with an increased occurrence of GDM (OR = 1.73, 95% CI: 1.10, 2.74) because of environmental contaminants [42]. In stark contrast, however, in the PREWICE II cohort study, Tryggvadottir EA et al., reported a higher median concentration of total alkylresorcinols of plasma as a whole-grain consumption biomarker in pregnancies women plasma without GDM rather than women diagnosed with GDM (209 nmol/L vs. 163 nmol/L, respectively; *p* < 0.001) [41]. The possible mechanism might be that whole-grain diet contained fiber and phytochemical components increased gut health, and improved glycemic response [23, 43].

Meanwhile, one study that has prospectively examined the correlation of fruit intake during pregnancy with GDM incidence, suggested that fresh fruit intake is inversely associated with the risk of GDM [44]. However, fruits were not been categorized in detail, which might result in misinformation about fruit type.

We are unaware of published prospective studies which assessed the DPI in relation to GDM. Pregnancy outcomes in relation to GDM adversely have imposed an immense burden on the global health system [45]. Hence, prevention and management of GDM should be getting as a high priority straight. This study is the largest to date to provide data that has investigated the correlation between DPI and GDM risk.

In this study, several strengths and limitations were present. There are no cohort studies have used propensity scores to evaluate the association between DPI and GDM which can preclude bias related to potential confounding variables. The propensity scoring implementation can control confounding by balancing covariates between exposed and non-exposed groups [46].

Moreover, strong recall bias may present through dietary assessment tool which assessed with FFQ. However, the use of a validated FFQ to collect dietary intake information, and standardized clinical assessments, as well as the prospective large sample size setting in this study can rule out mentioned bias. In this work, Iranian population with the same ethnicity diversity were recruited in order to limiting generalizability.

Inheritance limitation of DPI such as failing to add up non-caloric phytochemical-rich foods like green and black tea and spices should be considered. Furthermore, we failed to conduct dietary questionnaires during the early or pre-pregnancy which can interpret the relationship between DPI and GDM more clearly. Previous data reported that dietary patterns are not, however, varied during pregnancy [47].

## Conclusion

In summary, according to our finding, this prospective cohort study among pregnant women suggest that the DPI has no impact on GDM. More research is needed to determine the exact association between DPI and GDM.

## Data Availability

The datasets used and analyzed during the present study are available from the corresponding author upon reasonable request.

## Abbreviations

CI: Confidence interval
DAGs: Directed acyclic graphs
DPI: Dietary phytochemical index
FFQ: Food frequency questionnaires
GDM: Gestational diabetes mellitus
GBM: Generalized boosted model
IPW: Inverse probability weighting
IPTW: Inverse probability weight
IQR: Interquartile range
PS: Propensity scores
RR: Risk ratio
SD: Standard deviation
TLGS: Tehran Lipid and Glucose Study
